# Aneurysms—from traumatology to screening

**DOI:** 10.3109/03009730903518480

**Published:** 2010-04-07

**Authors:** David Bergqvist

**Affiliations:** Department of Surgical Sciences, Section of Vascular Surgery, Uppsala University Hospital, UppsalaSweden

**Keywords:** Aneurysm, atherosclerosis, surgery

## Abstract

This paper deals with aneurysmal disease, primarily when localized in the abdominal aorta. It is based on the Olof Rudbeck lecture 2009. Aneurysm is a localized widening of an artery, and its definition has become an important issue today when the disease is in focus for screening programmes. Aetiology and pathogenesis are still poorly understood, but a genetic component determining the strength of the aortic wall is important, and there is a strong male dominance. Historically, several attempts have been made to treat the disease, but reconstructive treatment has been possible only since 1951, in an increasing number of cases performed endovascularly. By early detection through screening, and thereby the possibility to treat before rupture, it has now become possible to decrease the total mortality from the disease in the population.

## Introduction

Aneurysmal disease continues to fascinate people––both professionals and laymen. There are certainly several reasons, but two important ones are that in the majority of cases the disease is asymptomatic but with an inherent risk to rupture, when it becomes dramatic and often ends with the patient's death––from no symptoms to a life-threatening condition within minutes to hours. Intuitively it therefore seems logical to detect the aneurysm and treat it before rupture. This paper will review the aneurysmal disease from a historical perspective with special emphasis on aneurysms in the abdominal aorta (triple A or AAA). The term abdominal aortic aneurysm is a linguistic mixture of Greek (*aneurynein*, to widen; *aeirein*, to heave) and Latin (*abdomen*, stomach).

## Definition

**Figure 1.**
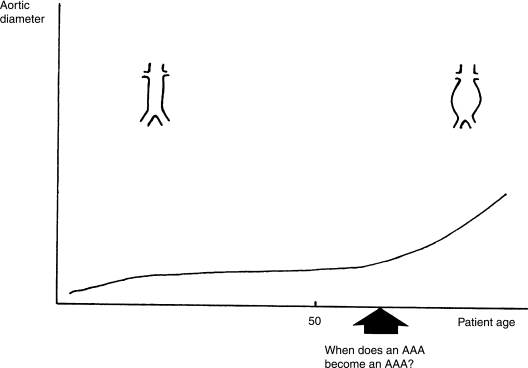
Schematic drawing of the aortic diameter during a lifetime.

There have been various definitions over time from the morphological appearance of a bulging artery to diameter measures in absolute terms and to aneurysm diameter in relation to the patient's non-aneurysmal aorta or to an ideal diameter obtained from nomograms (1). The definition was hardly a problem previously, when aneurysms were diagnosed because of symptoms, when they were large and therefore easily diagnosed even clinically. The definition has become an important issue when screening is discussed, where aneurysms of all sizes are detected and also very small ones have to be dealt with (Figure 1).

## Before 1900

From antique times until the 1800s the majority of aneurysms were traumatic in origin, and they were also the easiest to diagnose (a review of history in more detail in reference (2)). Already in the Synagogue Medica, Oribasius from Pergamon (325–403 AD) made the important classification into true (verum) and false (spurium) aneurysms in writing ‘there are two types of aneurysms: the first is due to dilatation of the arteries and the second is caused by rupture of the artery emptying blood into the tissues. When an aneurysm is due to dilatation the form is cylindrical, while the one caused by injury is round.’

One important subgroup of false aneurysms was those caused by blood-letting, when the artery was mistakenly punctured instead of the vein, i.e. an early type of iatrogenic vascular injuries. One such a case is described in detail by Olof Acrel in his ‘*Chirurgiske Händelser*’ from 1759 (3). The case was complicated, with infection leading to heavy haemorrhage and the patient's death, despite ligation undertaken by Acrel. The complication was well known with a couple of cases every year at the ‘Serafimer lazarettet’ where Acrel was the head, and the consensus among surgeons at the time was to ligate and rely on the collateral circulation to avoid ischaemia.

Another type of trauma was probably the cause of an aneurysm reported by John Hunter. He described a popliteal aneurysm in a coachman with repeated trauma to the popliteal fossa. It is interesting to note that before ligating such aneurysms Hunter had carried out animal experiments in deer belonging to the royal family in Richmond Park. He ligated the external carotid artery in the neck resulting in chilling of the antlers, but in a few days they had normal temperature again and by injecting coloured resin into the carotid artery he could visualize and demonstrate the collateral circulation. Obviously, we do not know whether the popliteal aneurysm of the coachman was post-traumatic or if it had an infectious cause such as lues, or perhaps a combination.

During the second half of the 1800s there were some theories on the cause of aneurysms, such as long-standing respiratory pauses, excessive copulation, and wearing too tight collars as was usually the case in army soldiers. By and by, however, two other explanations became prevalent, namely infection and atherosclerosis. Although the mechanism behind the infection was not clear, Sir William Osler, among others, described what he called mycotic aneurysms, so named because of the often bizarre and mushroom-like morphologic appearance of the aneurysm. As mentioned above, one cause of mycotic aneurysm was syphilis, often localized in the thoracic aorta and a frequent killer when rupture occurred. Arteriosclerosis as a cause of aneurysms was suggested because of the microscopic finding of arteriosclerosis in the aneurysmal wall. In German text-books there was a differentiation between ‘*stenosierende und dilatierende Atherosclerose*’, indicating a common aetiologic background to occlusive and aneurysmal disease!

The treatment of aneurysms until 1900 was basically ligature, often on both sides of the aneurysm. This technique was also used to treat an abdominal aortic aneurysm by Sir Astley Cooper in 1818, a dramatic and large operation at the time, although the patient survived only a couple of days. A more reconstructive way of thinking was introduced by Rudolph Matas in New Orleans, when he described his technique of aneurysmorrhaphy (first used 1888 in a patient with a post-traumatic brachial aneurysm) (4). The philosophy was to obliterate the aneurysmal sack leaving a channel in the middle for the blood to circulate.

## 1900 –1950

Around the end of the 19th century, there were a lot of activities of importance for understanding and treatment of vascular diseases, which means also for aneurysmal disease. The Nobel Prize was awarded for the detection of X-rays. Only a few years after the original report in 1896 it was used for angiography. Anaesthesiology developed, making increasingly advanced operations possible. Various graft materials were tried to replace blood vessels. For example, Abbe in New York (1896) used glass tubes experimentally in the aorta of cats: functioning but hardly practical. To replace a popliteal aneurysm in a patient, Goyanes in Lisboa used a reversed vein. Blood transfusion became practically possible when blood groups were described by Landsteiner, who was also awarded the Nobel Prize. The third Nobel laureate during this period of importance in this context was Alexis Carrel, who systematically described the basic vascular surgical technique, important for all types of vascular reconstructions and organ transplantations.

During this period not much happened regarding the pathophysiological understanding of the aneurysmal disease, the dominating cause being considered arteriosclerosis.

Regarding treatment, ligature was still used, although this had as a prerequisite an adequate collateral circulation, which was not always the case. Aneurysmorrhaphy had come to stay, and among others Matas himself in 1942 reported a large personal experience of treating aneurysms. Another therapeutic attempt was to induce thrombotic occlusion, most often done by inserting a foreign material into the aneurysmal sack, such as metallic wires, sometimes also used to stimulate coagulation by an electric current. More or less sophisticated instrumentation was invented to reach the goal of thrombotic occlusion. Unfortunately intra-aneurysmal thrombosis did not prevent rupture.

Knowing that the size of the aneurysm and its expansion were important to predict rupture, a logical treatment step was to try to stabilize the wall and prevent expansion. This was done using various types of material, both synthetic (such as plastic films and cellulose) and biological (such as fascia). Whether this method slowed down the expansion rate is not known, but it did not prevent rupture. The most famous illustration of this is Albert Einstein, whose aneurysm was wrapped in 1948 by the well known New York surgeon Rudolph Nissen, but Einstein's death in 1955 was due to rupture.

## 1950 –1998 (United Kingdom Small Aneurysm Trial)

Around 1950 the professional discussion had started whether or not it was possible to reconstruct the aorta instead of resecting it, which had been recommended and used by René Leriche in case of occlusive disease. Such a reconstruction with a homograft was carried out by Oudot in 1951, and in the same year patients with AAA were also surgically treated. The first two can be attributed to Norman Freeman in San Francisco, the first patient bleeding to death shortly after the operation, the second with long-term survival (5). Freeman used an inlay technique with a vein graft to obtain circulation. A month later, Dubost (6) in Paris resected an AAA using a homograft for reconstruction in a similar way as Oudot (7). Soon synthetic materials were used for the reconstruction (8), and operation for rupture with survival was introduced (9). Operating on patients with an asymptomatic disease to prevent a statistical risk for rupture had to show very low mortality and complication rates. This surgical method rapidly spread, and publications soon appeared with a postoperative survival only slightly inferior to that of a matched normal population.

The next step in how to treat patients with AAA came in 1986 when Nicolai Volodos in Ukraine used stent-graft reconstruction, and in 1988 he used a similar method to treat thoracic aortic aneurysm. Unfortunately his first two publications were in Russian and therefore largely unknown outside the Soviet Union. A similar method was used by Juan Parodi (10), and although Volodos published an English report in 1991 in Vasa (11), it was the paper by Parodi in combination with his move from Argentina to the US which had a great impact on how to treat AAA endovascularly. The method was attractive avoiding a large intraperitoneal operation, and the technology spread rapidly although not properly evaluated.

**Figure 2.**
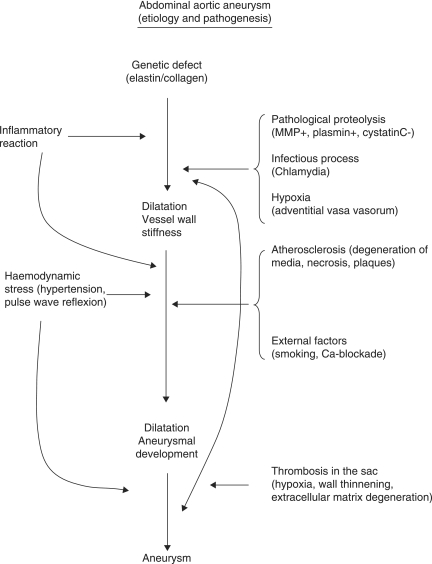
Factors influencing the development of an aortic aneurysm.

During this period data accumulated questioning that AAA is a manifestation of atherosclerosis. Probably the process is more complex, atherosclerosis coming in rather late in the natural course of the aneurysmal disease. It could well be that the development of aneurysm is a common pathologic response to various types of influences on the arterial wall, some of them being summarized in Figure 2. In many cases it has become evident that there is an inborn defect in the strength of the aortic wall (collagen and/or elastin), with an inherited pattern. So sons of patients with an AAA have an exceptionally high prevalence of AAA (12). Better understanding of the biology of AAA will certainly influence how to design studies in the future with the aim to prevent AAA or at least to slow down expansion rate once they have occurred.

**Figure 3.**
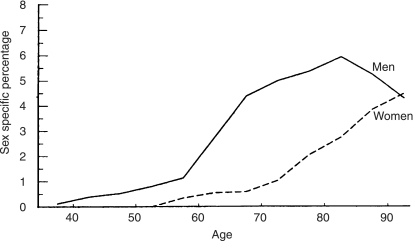
Sex-specific prevalence of abdominal aortic aneurysm confirmed by necropsy (from Bengtsson et al. 1992 with permission (28)).

Data also started to appear on the epidemiology of AAA. Age and gender are the most important factors influencing the prevalence (Figure 3). There may also be some geographical differences (Table I). Table II shows the most important risk factors for AAA. Some of them are also risk factors for complications and mortality in relation to surgical treatment. Although there has been an increase in elective surgery for AAA, the rate of rupture has not decreased to a similar extent or even increased (13), indicating a problem in selecting the correct patients for surgery.

**Table I.** Frequency of aneurysms in the abdominal aorta (AAA) in the general male population (ultrasonographic screening).

					Prevalence (%)	
Author	Reference	Country	Attendance rate (%)^a^	Age (years)	Total	> 50 mm
Bengtsson	(29)	Malmö, Sweden	75	74	10.7	2.2
Scott	(30)	UK	59	65–80	7.6	1.0
Lederle	(17)	USA	30	50–79	4.6	0.5
Wanhainen	(27)	Norsjö, Sweden	91	65–75	17.3	4.8
Wanhainen	Unpublished	Uppsala, Sweden	84	65	3.3	0.5

^a^Number attending the screening of those invited (in per cent).

**Table II.** Risk factors for abdominal aortic aneurysm.

Male gender
Age
Smoking
Family history
Occlusive arterial disease

## From 1998 onwards

This period can also be called the era of evidence-based medicine, because during this period some of the very important randomized trials in vascular surgery have been published and certainly influenced our ways of treating patients with vascular disorders.

The aneurysmal size at which to contemplate surgical treatment has been set to 55 mm (14,15). In patients with an aneurysm diameter of 4.0–5.5 cm in diameter, surveillance and early prophylactic elective surgery have similar survival benefits. However, after 8 years, total mortality was lower in the early-surgery group, probably due to beneficial life-style changes, especially concerning smoking (16). Similar results came out from the ADAM trial (Aneurysm Detection And Management) (17), and results from the two studies have been meta-analysed in a Cochrane review (18).

In 2004 two randomized studies were published comparing open surgery with endovascular repair (EVAR) in abdominal aneurysms larger than 5–5.5 cm in diameter. Both showed a short-term benefit for EVAR but concluded that long-term results had to be awaited before giving firm recommendations (19,20). In a Medicare cohort study of 45,660 patients, these early results have been verified, but late re-interventions were more common after endovascular repair (21). Further support for early benefit in EVAR-treated patients is given in a planned interim analysis from an on-going American veterans administration (VA) trial (22).

The World Health Organization criteria for screening to be considered are summarized in Table III. Most of them now seem to be fulfilled when it comes to AAA (23). The most important ones are that asymptomatic adult subjects can harbour potentially life-threatening disorders, that the disease of interest can be detected at an early stage with a methodology that is safe and accepted, and that discovery of the disease can lead to some form of treatment, which positively influences the patients' survival and/or morbidity. Large randomized trials on screening have now been performed and also meta-analysed (24), showing that aneurysm-related mortality can be reduced in the population by introducing ultrasonographic screening. The mortality benefit seems to be maintained at least for 10 years (25). A recent publication from the Swedish Board of Health Technology Assessment recommends general screening of 65-year-old males, and this is also cost-effective (26). This policy has been introduced in several Swedish counties, and at the moment of preparing this article screening covers about 80% of the Swedish population. Whether or not to screen 70-year-old females is dealt with in a research project in Sweden at the moment.

**Table III.** World Health Organization criteria for surveillance programmes.

The disease should be an important health problem. The disease should have a high prevalence. The biological behaviour must be shown. The surveillance/screening test must have a high sensitivity and specificity. A method of treatment should be available. The screening programme must be cost-effective.

General introduction of screening will have some consequences. A number of aneurysms will be detected, some of them of a size where surgery can be strongly recommended. This will hopefully decrease the rupture rate and thereby the need for emergency surgery, turning the acute cases into elective ones. Some problems will occur when detecting small aneurysms, where the subjects will turn from being healthy individuals to patients with a potentially serious disease. They have to be included in a follow-up programme and some, in case of increase in size, may be offered surgical treatment. This obviously also has ethical implications. One advantage (on a population basis) is that the cohort of patients with small aneurysms can be offered taking part in various randomized trials investigating substances with the potential to slow down aneurysmal expansion rate with the final aim to avoid the need for surgery and the risk of rupture (Table IV).

**Table IV.** Substances of interest to analyse whether or not there is a favourable influence on expansion rate of abdominal aortic aneurysms.

Statins
Angiotensin-converting enzyme inhibitors
Macrolides
Cyclo-oxygenase inhibitors
Indomethacin
Tetracyclines
Vapamycin
Doxycycline
Roxithromycin
Azithromycin

## Concluding remarks

That AAA is a disease known in the population as a serious one is reflected in two recent Swedish publications. One is the autobiography ‘Viewing back’ from 2008 by the Professor of History of Ideas in Gothenburg, Sven-Erik Liedman (31). He is writing about his grandfather: ‘He was an optimist to the final end. When he was dying I sat by his side. The aorta had ruptured when he was working in his garden with stabilizing pea plants and in his unconsciousness he continued with that.’ The other citation is from ‘Dorés Bibel’ (2005) (32) by the member of the Swedish Academy Torgny Lindgren, illustrating a common knowledge in the population of the Norsjö community, a knowledge that was verified by ultrasonographic screening by Anders Wanhainen (27): ‘We inhabitants in Norsjö have a peculiar kind of aneurysms in our abdominal cavity, I said. They tend to rupture, that is what is killing us. This one was exceedingly larger than a varicosity, Manfred said.’

This fascinating history of aneurysmal disease has brought us to today's screening programmes, which––if they become largely accepted––will for the first time give us a very good way of decreasing the total mortality of aneurysmal disease within the population, based on scientific evidence.
